# Food access, mobility, and transportation: a survey and key informant interviews of users of non-profit food hubs in the City of Vancouver before and during the COVID-19 crisis

**DOI:** 10.1186/s12889-021-12434-9

**Published:** 2022-01-04

**Authors:** Daniel Rajasooriar, Tammara Soma

**Affiliations:** School of Resource and Environmental Management, Simon Fraser University, Burnaby, Canada

**Keywords:** Food security, Food access, Mobility, Transportation, Food assets, Non-profit food hubs, Food system planning, COVID-19

## Abstract

**Background:**

In the City of Vancouver, Canada, non-profit food hubs such as food banks, neighbourhood houses, community centres, and soup kitchens serve communities that face food insecurity. Food that is available yet inaccessible cannot ensure urban food security. This study seeks to highlight food access challenges, especially in terms of mobility and transportation, faced by users of non-profit food hubs in the City of Vancouver before and during the COVID-19 crisis.

**Methods:**

This study involved an online survey (*n* = 84) and semi-structured follow-up key informant interviews (*n* = 10) with individuals at least 19 years old who accessed food at a non-profit food hub located in the City of Vancouver more than once before and during the COVID-19 crisis.

**Results:**

88.5% of survey respondents found food obtained from non-profit food hubs to be either very or somewhat important to their household’s overall diet. In their journey to access food at non-profit food hubs in the City of Vancouver, many survey respondents face barriers such as transportation distance/time, transportation inconveniences/reliability/accessibility, transportation costs, line-ups at non-profit food hubs, and schedules of non-profit food hubs. Comments from interview participants corroborate these barriers.

**Conclusions:**

Drawing from the findings, this study recommends that non-profit food hubs maintain a food delivery option and that the local transportation authority provides convenient and reliable paratransit service. Furthermore, this study recommends that the provincial government considers subsidizing transit passes for low-income households, that the provincial and/or federal governments consider bolstering existing government assistance programs, and that the federal government considers implementing a universal basic income. This study emphasizes how the current two-tier food system perpetuates stigma and harms the well-being of marginalized populations in the City of Vancouver in their journey to obtain food.

## Background

Food security has been defined as “a situation that exists when all people, at all times, have physical, social and economic access to sufficient, safe, and nutritious food that meets their dietary needs and preferences for an active and healthy life” ([1] p49). For its part, the United Nations has been advocating for the end of hunger and the achievement of food security as one of the Sustainable Development Goals [2]. Despite attempts to end hunger and achieve food security, there is a growing number of people affected by food insecurity. Globally, 2 billion people were affected by moderate or severe food insecurity in 2019, up from 1.9 billion in 2014 [2]. Although some may be tempted to think that food insecurity is only prevalent in developing countries, it is also prevalent in developed countries such as Canada. In Canada, 4.4 million people were living in a household affected by marginal, moderate, or severe food insecurity in 2017–2018, up from 3.9 million in 2011–2012 [3, 4]. The COVID-19 crisis has only exacerbated the issue of food insecurity globally and in Canada [2, 5]. During the COVID-19 crisis, 14.6% of Canadians were living in a household where there was moderate or severe food insecurity during April–May 2020, up from 10.5% during 2017–2018 [5].

The International Covenant on Economic, Social, and Cultural Rights asserts the right to adequate food and the fundamental right to be free from hunger [6]. As a state party to the covenant, which has been in effect since 1976, the Government of Canada has “the obligation to respect, promote, and protect and to take appropriate steps to achieve progressively the full realization of the right to adequate food” ([7] p6). Although the Government of Canada attempts to secure the right to adequate food through social assistance and unemployment insurance programs, these programs have been inadequate. For example, 60.5% of households in Canada where the main source of income is social assistance programs face marginal, moderate, or severe food insecurity [4]. Meanwhile, 32% of households in Canada where the main source of income is employment insurance or workers’ compensation face marginal, moderate, or severe food insecurity [4].

The inadequacy of these programs has made it necessary for food banks and other non-profit organizations to fill the gap [8]. Food banks began to proliferate in Canada in the early 1980s as what was meant to be a temporary response to needs arising from the economic recession of that period [8]. Yet, demand for food banks did not abate as the economy improved because there were social policy reforms at the federal and provincial/territorial levels that resulted in reduced benefit levels, more restrictive eligibility criteria for social assistance and unemployment insurance programs, as well as retractions in investments in social housing [8]. The trend that has persisted since then is that the bulk of food bank clientele have been reliant on welfare and other social assistance programs [8].

While food banks and other non-profit organizations can certainly support immediate and temporary needs, they are not the best solution in terms of securing the right to adequate food, nor are they the best solution in terms of supporting nutrition security [9]. For example, food banks and other non-profit organizations tend to have a selection of food that does not satisfy nutrition standards and tend to run out of food [10]. Furthermore, food banks and other non-profit organizations typically do not cater to special diets, whether due to a medical condition, an ethical or religious belief, or a preference [10].

Currently, solutions to address urban food insecurity in Canada are focused on improving existing food assets and/or growing the number of food assets in cities. Baker notes the central role of food assets in promoting food security [11]. The definition of food assets is:

the local food infrastructure that maintains food-secure communities and regions – farms, processing and distribution capacity, food enterprises, markets, retailers,. .. urban farms, community gardens, community kitchens, student nutrition programmes, emergency food distribution and community food organizations and centres” (11 p266).

This study focuses on the subset of food assets that come under the umbrella term of *non-profit food hubs* in the City of Vancouver, Canada. Examples of non-profit food hubs include food banks, neighbourhood houses, community food centres, and soup kitchens. Drawing from an online survey (*n* = 84) and semi-structured follow-up key informant interviews (*n* = 10), this study evaluates access to food at non-profit food hubs in the City of Vancouver before and during the COVID-19 crisis, especially as it relates to mobility and access to transportation. The study addresses the following research questions: 1) Which individuals and families access non-profit food hubs in the City of Vancouver? 2) How did/do they access these non-profit food hubs before/during the COVID-19 crisis? and 3) What were/are the barriers to accessing food at these non-profit food hubs before/during the COVID-19 crisis.

### Food security and non-profit food hubs in Canada

The important role that food banks and other non-profit organizations in Canada play in terms of attempting to secure the right to adequate food has been evident through the COVID-19 crisis and has been recognized by the Government of Canada. When many vulnerable Canadians were facing the impacts of the COVID-19 crisis in April 2020, the Government of Canada announced $100 million in funding toward an Emergency Food Security Fund for food banks and other non-profit organizations so that they could make sure people could get the food they need [12]. In October 2020 and August 2021, the Government of Canada announced additional rounds of $100 million in funding [12]. The funds were disbursed to several key organizations: Food Banks Canada, Second Harvest, Community Food Centres Canada, Breakfast Club of Canada, Salvation Army, and La Tablée des Chefs [12]. Other organizations interested in receiving funding were required to reach out to those key organizations [12].

It is important to consider how the Government of Canada has addressed food insecurity among Indigenous communities during the COVID-19 crisis through a separate Indigenous Community Support Fund [12, 13]. Given the existence of both an Emergency Community Support Fund and the Emergency Food Security Fund which several key non-Indigenous organizations have direct access to, Levi and Robin argue that it would be beneficial for Indigenous-led and/or Indigenous-focused organizations to have similar direct access to the Emergency Food Security Fund [12, 14, 15]. Though food banks and other non-profit organizations in Canada play an important role in terms of attempting to secure the right to adequate food, rural and remote Indigenous communities are harder to reach and have different food-related needs (e.g., cultural) that would be better met by Indigenous-led and/or Indigenous-focused organizations.

### Food security and the City of Vancouver

The *Poverty Reduction Plan, What We Heard: Phase 1* report published by the City of Vancouver highlights significant food access challenges for low-income individuals and families [16]. The report highlights that difficulty in accessing food from spaces such as food banks and charities can lead to further physical and psychological harm which in turn sustains oppressive systems of poverty [16]. Since the report by the City of Vancouver was published prior to the COVID-19 crisis, this study provides valuable insight into how food access challenges have played out during the COVID-19 crisis [16]. As O’Hara and Toussaint note, the COVID-19 crisis has disproportionately impacted food access for those who are already food insecure [17]. Furthermore, a report published by the City of Vancouver on populations impacted by the COVID-19 crisis found that people from Indigenous, racialized, and immigrant communities are disproportionately impacted by the inability to meet basic needs such as food and shelter [18].

### Food security and food sovereignty

There are four dimensions of food security, namely, availability, access, utilization, and stability [19]. The dimension of availability addresses the physical availability of food, the dimension of access addresses physical and economic access to food, the dimension of utilization addresses the nutritional adequacy of food intake, and the dimension of stability addresses whether the other dimensions are stable over time [20]. The dimensions of availability, access, and utilization are inherently hierarchical since availability is necessary but not sufficient to ensure access and since access is necessary but not sufficient for effective utilization [21].

One factor that contributes to physical access to food is the spatial proximity of food assets. The importance of this factor has given rise to the concept of food deserts. Food deserts have been defined as areas lacking access spatially to healthy foods, like fruits and vegetables, as well as other nutritious food options [22]. However, increasing the availability of food assets alone is not sufficient. While there may be food assets with affordable and adequate food near an individual’s or family’s place of residence, they might not be able to access these food assets during the food asset’s hours of operation due to the need to travel for work and/or complete other daily tasks [23, 24].

Another factor that contributes to physical access to food is mobility and access to transportation. If individuals or families have mobility issues and/or a lack of access to transportation, they may not be able to access food assets, even if they are spatially proximate. In terms of mobility, research indicates that having a disability, including those that do not relate to mobility, often results in decreased access to food and an increased chance of food insecurity [25]. In terms of access to transportation, many cities around the world, and especially those in the North American context, have a built environment that is centred around the use of automobiles [26, 27]. It has been found that the degree to which households without automobiles are able to make alternative travel arrangements to go shopping for food is far more influential in terms of physical access to food than the factor of spatial proximity of food assets [28]. If public transportation is not available or readily accessible, then households without automobiles may go grocery shopping less frequently and may access food assets such as nearby convenience stores where there are limited options in terms of quality food that meets dietary needs and preferences [29, 30].

In terms of economic access to food, income is the driving factor. Low-income individuals and families, especially children, are more likely to face food insecurity [4, 31]. These individuals and families are vulnerable because they spend a large share of their income on food [32]. They may have to cut down on the quantity and/or quality of food they obtain to save money and thereby not meet their dietary needs and preferences [29]. Beyond income, the cost of food and/or the cost of transportation can also impact a low-income individual’s or family’s economic access to food.

It is important to note that many scholars have been critical of the concept of food security and have argued that we should move toward the concept of food sovereignty [33–35]. Food sovereignty has been defined as “the right of peoples to healthy and culturally appropriate food produced through ecologically sound and sustainable methods, and their right to define their own food and agriculture systems” ([36] p9). Some scholars note that the juxtaposition of food security and food sovereignty may be more confusing than helpful when it comes to policy dialogue on the food system because it suggests a false dichotomy [34, 37]. However, where food sovereignty differs is the focus on people being able to define their own food and agricultural systems. As such, a food sovereignty approach does more to challenge a top-down or charity-model approach to tackling food insecurity, where community members are not provided with extensive freedom of choice (e.g., food banking). By addressing the root causes of food insecurity, food sovereignty approaches have been found to positively influence food security and nutrition outcomes [38].

## Methods

This study has secured research ethics approval from the [name of the University] Research Ethics Board. Informed consent was obtained from all participants before commencing the study. The study utilizes quantitative and qualitative methods of data collection to build a better understanding of the intersection of access to transportation and access to food for users of non-profit food hubs in the City of Vancouver. There are two components to this study, namely, an online survey and key informant interviews. Interview participants were only recruited from among the survey respondents. The purpose of the key informant interviews was to provide opportunities for survey respondents to speak freely about their experiences. In terms of inclusion/exclusion criteria, this study elicited responses from individuals at least 19 years old who accessed food at a non-profit food hub located in the City of Vancouver more than once before and during the COVID-19 crisis.

The online survey opened on the 22nd of June 2020 and closed on the 31st of August 2020. Survey respondents were recruited by disseminating a link to the online survey. A link to the online survey was disseminated to those who access food at a non-profit food hub with the help of some non-profit food hubs in the City of Vancouver and the BC Poverty Reduction Coalition. The link was disseminated via email, social media, and the BC Poverty Reduction Coalition’s website. 84 completed survey responses were received. On average, survey respondents took approximately 12 min to complete the online survey. Survey respondents were offered a $10 gift card in exchange for their involvement.

For the qualitative component of the study, key informant interviews were conducted between the 22nd of July 2020 and the 31st of October 2020. Interview participants were recruited from among survey respondents by having a question in the online survey asking survey respondents if they were willing to be contacted for a key informant interview. Survey respondents who indicated a willingness to be contacted for a key informant interview were contacted via email and/or phone to set up an interview. They were given a choice of having the interview done over the phone or a Zoom meeting. All 10 interview participants chose to complete phone interviews. On average, interview participants took approximately 30–60 min to complete the key informant interview. Interview participants were offered a $20 gift card in exchange for their involvement. Quotes from the interview participants have been anonymized with the use of pseudonyms.

## Results

### Demographics

A profile of survey respondents is found in Table 1. In terms of age, 45.2% of survey respondents (*n* = 84) were 25 to 34 years old and 19% were 19 to 24 years old. As mentioned in the limitations section, a large proportion of survey respondents being young adults may have been influenced by how an online survey may not be accessible to those older adult demographics who are not proficient in the use of the technology. In terms of gender identity, whereas 58.8% of survey respondents (*n* = 80) were female, 40% were male. In terms of ethnic background, 74.4% of survey respondents (*n* = 82) identified as White, 12.2% identified as Indigenous, and many identified as various minorities. As mentioned in the limitations section, although a large proportion of survey respondents identified as White, this does not align with the reality that Indigenous people and visible minorities, particularly Black people, disproportionately experience food insecurity in Canada [4]. This discrepancy and others in this study may be attributed to this study’s limitations. Moreover, the discrepancies may be impacted by how the category of non-profit food hubs is much broader than the category of food banks and how the City of Vancouver is a much narrower study area than British Columbia. In terms of highest level of formal education, 9.6% of survey respondents (*n* = 83) had less than a high school diploma, 8.4% had a high school diploma or an equivalent, 53% had some post-secondary education or a post-secondary certificate, and 28.9% had a degree at the bachelor’s level or above. Although a large proportion of survey respondents seem to be well-educated, this does not align with the reality that food insecurity is more prevalent in households with lowers levels of education [4]. Whereas 11% of survey respondents (*n* = 82) were the sole individual in their household, 89% of those who access non-profit food hubs in the City of Vancouver were from multi-person households.

**Table 1:** Profile of survey respondents

Item	Category	Percentage (%)
**Age (*n*** **= 84)**	19–24	19.0
	25–34	45.2
	35–44	11.9
	45–54	13.1
	55–64	6.0
	65–74	1.2
	75 and over	3.6
**Gender Identity (*n*** **= 80)**	Female	58.8
	Male	40.0
	Other	1.3
Ethnicity* (*n* **= 82)** *Survey Respondents Could Select Multiple Ethnicities	White	74.4
	East and Southeast Asian	11.0
	*Chinese*	*1.2*
	*Filipino*	*3.7*
	*Southeast Asian*	*1.2*
	*Japanese*	*1.2*
	*Korean*	*3.7*
	South Asian	4.9
	Indigenous	12.2
	Latin American	3.7
	West Asian and Arab	2.4
	*West Asian*	*2.4*
	*Arab*	*0.0*
	Black	6.1
**Highest Level of Formal Education (*n*** **= 83)**	Less than a high school diploma	9.6
	High school diploma or equivalent	8.4
	Some post-secondary education	26.5
	Post-secondary certificate/diploma	26.5
	Degree at the bachelor’s level	22.9
	Degree above the bachelor’s level	6.0
**Household Structure (*n*** **= 80)**	Two parents	43.8
	Single parent	15.0
	Couple with no dependent children	18.8
	Single person	11.3
	Other	11.3
Number of Households Members* (*n* **= 82)** *Including Survey Respondent	1	11.0
	2	18.3
	3	8.5
	4	13.4
	5 or more	48.8
Total Household Income* (*n* **= 81)** *Before taxes in the past 12 months, including any government assistance programs.	Less than $30 k	29.6
	$30 k to less than $60 k	35.8
	$60 k to less than $90 k	27.2
	$90 k or more	7.4

In terms of survey respondents’ household structures, it is beneficial to draw comparisons between this study’s findings and Food Banks Canada’s findings [39]. There is a large discrepancy between this study’s percentage of survey respondents (*n* = 80) from two-parent families with at least one dependent child (43.8%) and Food Banks Canada’s percentage of food bank clientele in British Columbia from two-parent families with at least one dependent child (15.4%) [39]. Furthermore, there is a large discrepancy between this study’s percentage of survey respondents from households with at least one dependent child (58.8%) and Food Banks Canada’s percentage of food bank clientele in British Columbia from households with at least one dependent child (31.1%) [39]. Additionally, there is a large discrepancy between this study’s percentage of survey respondents from single-person households (11.3%) and Food Banks Canada’s percentage of food bank clientele in British Columbia from single-person households (51.5%) [39]. In terms of the last two discrepancies, it is worth noting that the relevant findings from this study are consistent with how households in Canada with one or more dependent children are more likely to face household food insecurity than households in Canada without any dependent children [4, 40]. Looking to similarities, this study’s percentage of survey respondents from single-parent families (15%), all of whom were female, is similar to Food Banks Canada’s percentage of food bank clientele in British Columbia from single-parent families (15.7%) [39]. Furthermore, this study’s percentage of survey respondents who are couples with no dependent children (18.8%) is somewhat similar to Food Banks Canada’s percentage of food bank clientele in British Columbia who are couples with no dependent children (12.8%) [39].

In terms of survey respondents’ total household incomes (*n* = 81) in the past 12 months, 29.6% were less than $30 k, 35.8% were between $30 k and less than $60 k, 27.2% were between $60 k to less than $90 k, and 7.4% were $90 k or more. When reporting their total household income in the past 12 months, survey respondents were prompted to include any income from government assistance programs. As such, it is concerning that over 29.6% of survey respondents were making less than $30 k including any income from government assistance programs. Having a low income and/or high cost of living can cause immense financial stress for individuals and families. Although it may be surprising to note that 7.4% of survey respondents had a total household income of $90 k or more, those who indicated this total household income were from multi-person households. It is possible that they do not have access to household income. Furthermore, the category of non-profit food hubs does not cater exclusively to low-income individuals and families.

**Fig. 1:**
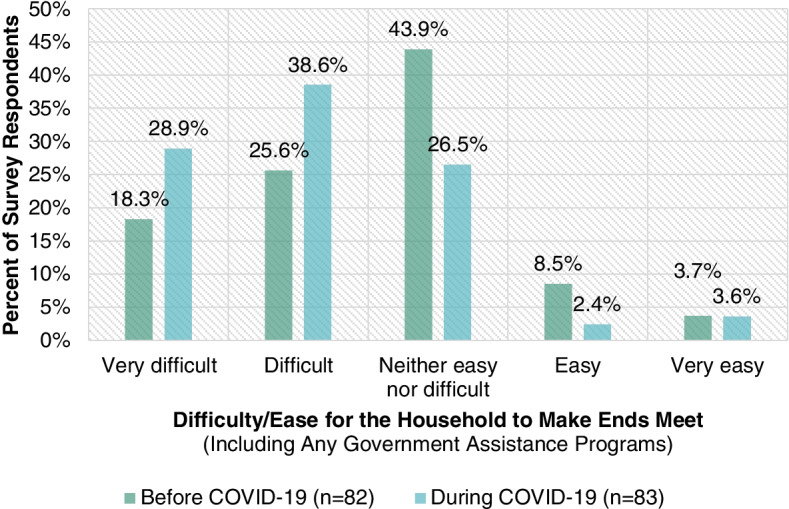
Difficulty/ease for survey respondents’ households to make ends meet

Fig. 1 highlights the difficulty or ease for survey respondents’ households to make ends meet, including any government assistance programs, before and during the COVID-19 crisis. Whereas 43.9% of survey respondents’ households found it very difficult or difficult to make ends meet including any government assistance programs before the COVID-19 crisis, 67.5% did during the COVID-19 crisis. This underscores how the COVID-19 crisis has exacerbated financial stress for low-income individuals and families.

### How non-profit food hubs are accessed

**Fig. 2:**
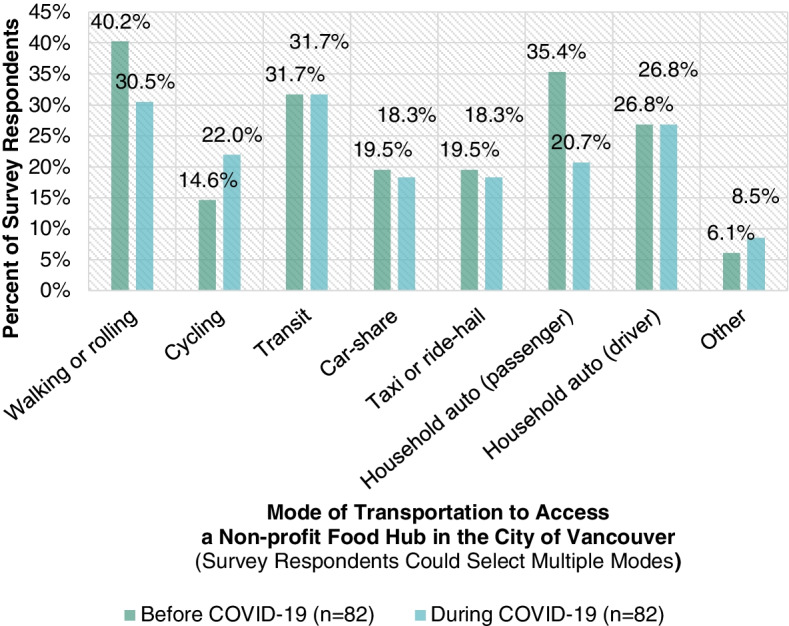
Modes of transportation used by survey respondents to access a non-profit food hub in the City of Vancouver

Figure 2 highlights the diverse modes of transportation used by survey respondents (*n* = 82) to access a non-profit food hub in the City of Vancouver before and during the COVID-19 crisis. Since survey respondents were given the opportunity to select multiple modes of transportation, it is not clear which mode of transportation has been the dominant mode of transportation used by survey respondents to access a non-profit food hub in the City of Vancouver. However, it is evident that a household automobile, walking or rolling, and transit were preferred modes of transportation to access a non-profit food hub in the City of Vancouver before and during the COVID-19 crisis. Since the COVID-19 crisis, fewer survey respondents have accessed a non-profit food hub in the City of Vancouver by walking or rolling and by a household automobile as a passenger, and more survey respondents have done so by cycling. Some survey respondents wrote-in additional modes of transportation such as HandyDART (the local paratransit system for people with disabilities) and the automobile of a friend. Based on the written-in modes of transportation for during the COVID-19 crisis, many survey respondents noted that the non-profit food hub in the City of Vancouver that they access had been delivering food to them. One survey respondent noted that they sold their household automobile during the COVID-19 crisis and now rely on transit and walking.

**Fig. 3:**
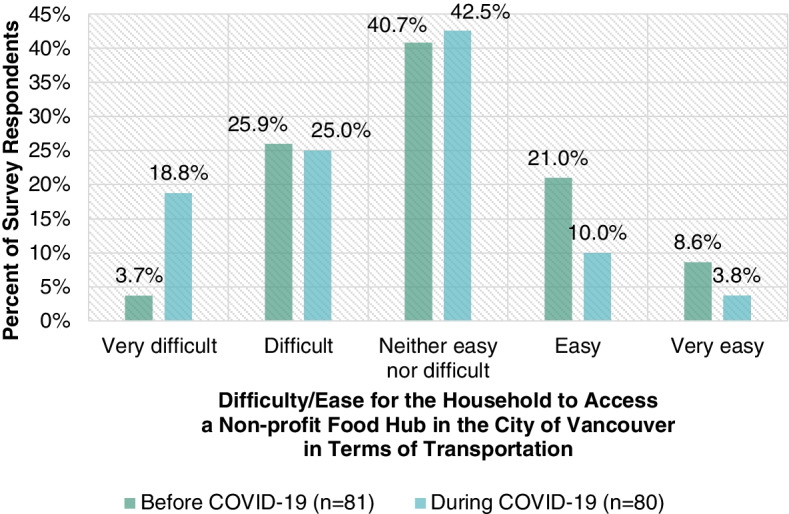
Difficulty/ease for survey respondents’ households to access a non-profit food hub in the City of Vancouver in terms of transportation

Fig. 3 highlights the difficulty or ease for survey respondents’ households to access a non-profit food hub in the City of Vancouver in terms of transportation before (*n* = 81) and during (*n* = 80) the COVID-19 crisis. Whereas 29.6% of survey respondents’ households found it very difficult or difficult to access a non-profit food hub in the City of Vancouver in terms of transportation before the COVID-19 crisis, 43.8% did during the COVID-19 crisis. Negative experiences in terms of transportation may be due to a host of factors including barriers such as including transportation accessibility, transportation costs, transportation distance, transportation inconveniences, transportation reliability, and transportation time. As discussed in the background section, having a very difficult or difficult experience in terms of transportation can deter an individual or family from accessing food [29]. Individuals and families may end up making less frequent trips to access food, thereby reducing the freshness and/or quality of the food they obtain [29].

**Fig. 4:**
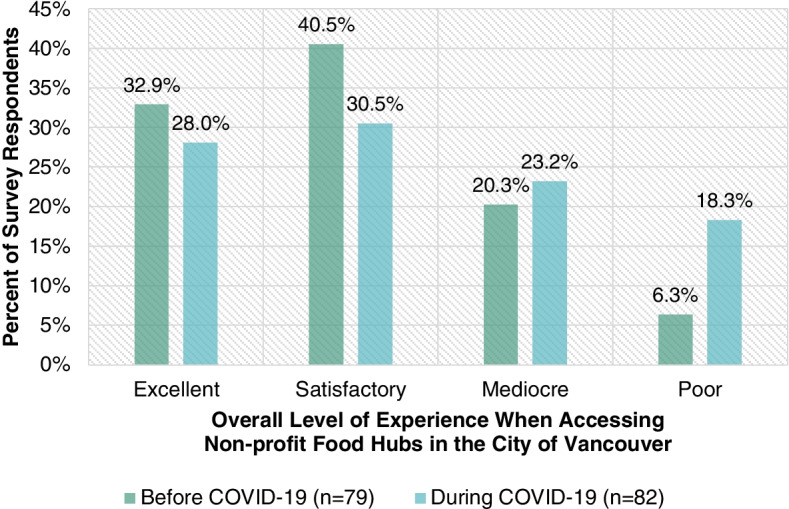
Overall level of experience survey respondents have when accessing non-profit food hubs in the City of Vancouver

Fig. 4 highlights the overall quality of the experience survey respondents had when accessing non-profit food hubs in the City of Vancouver before (*n* = 79) and during (*n* = 82) the COVID-19 crisis. Whereas 26.9% of survey respondents had a mediocre or poor experience when accessing a non-profit food hub in the City of Vancouver before the COVID-19 crisis, 41.5% did during the COVID-19 crisis. Negative experiences at non-profit food hubs may be due to a host of factors including barriers such as line-up times at non-profit food hubs and schedules of non-profit food hubs. As discussed in the background section, another factor may be how the food at non-profit food hubs is not always sufficient, nutritionally balanced, or otherwise adequate [10].

**Fig. 5:**
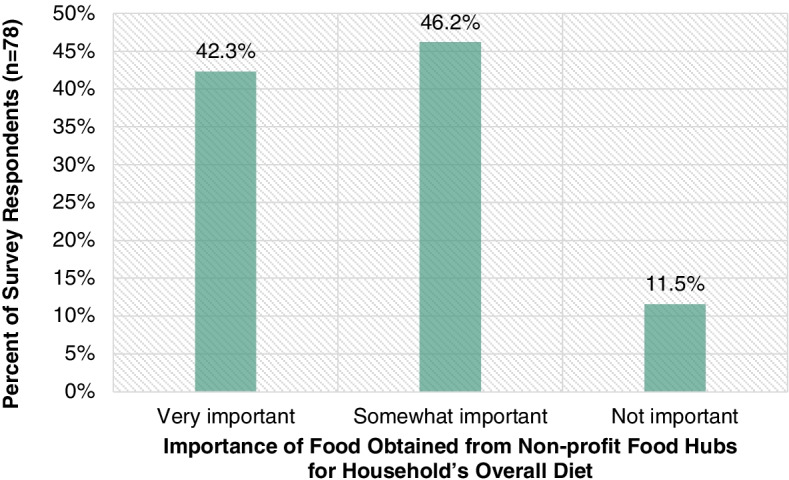
Importance of food obtained from non-profit food hubs for the survey respondents’ households’ overall diets

Figure 5 highlights the importance of foods obtained from non-profit food hubs for survey respondents’ households’ overall diets. 42.3% of survey respondents (*n* = 78) said that food obtained from non-profit food hubs is a very important part of their household’s overall diet, and 46.2% said it was a somewhat important part. Only 11.5% of survey respondents said that food obtained from non-profit food hubs is not an important part of their household’s overall diet. While the category of non-profit food hubs is broad and non-profit food hubs are not exclusive to low-income individuals and families, it is likely that only low-income individuals and families would consider food obtained from non-profit food hubs to be very important or somewhat important to their household’s overall diet. Therefore, the findings in Fig. 5 may underscore the important role that non-profit food hubs play in promoting economic access to food for low-income individuals and families [8].

### Barriers to accessing non-profit food hubs

**Fig. 6:**
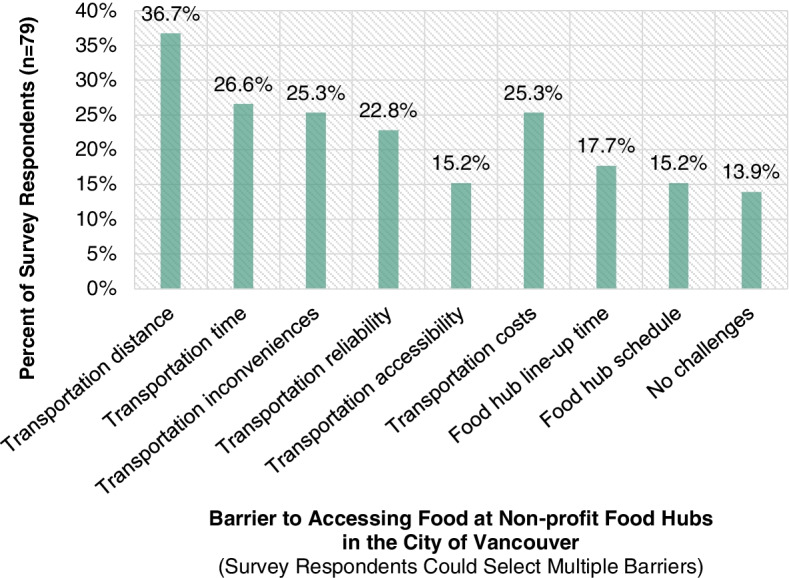
Barriers survey respondents face to access food at non-profit food hubs in the City of Vancouver

Figure 6 highlights the frequency of pre-identified barriers to accessing food at non-profit food hubs in the City of Vancouver that were felt by survey respondents (*n* = 79). The barriers were pre-identified by scanning through the *Poverty Reduction Plan, What We Heard: Phase 1* report published by the City of Vancouver which highlights significant food access challenges for low-income individuals and families [16]. Survey respondents were given the opportunity to select multiple barriers. Given connections between several of the barriers, some of them have subsequently been grouped. Transportation distance has been grouped with transportation time, and transportation inconveniences has been grouped with transportation reliability and transportation accessibility. When considering grouped barriers, the barrier of transportation distance/time was the biggest barrier that survey respondents identified with in terms of accessing food from a non-profit food hub in the City of Vancouver. Furthermore, when considering grouped barriers, the barrier of transportation inconveniences/reliability/accessibility was the second biggest barrier, the barrier of transportation costs was the third biggest barrier, the barrier of line-up times at non-profit food hubs was the fourth biggest barrier, and the barrier of schedules at non-profit food hubs was the fifth biggest barrier. Comments from interview participants corroborate the importance of addressing the preidentified barriers. In the following subsections, corroborating comments from interview participants are highlighted.

### Transportation distance and time

36.7% of survey respondents (*n* = 79) identified with the barrier of transportation distance and 26.6% identified with the barrier of transportation time. Transportation distance and transportation time were the barriers that were most identified with, first and second, respectively. The two barriers have been grouped because they are connected. When considering grouped barriers, the barrier of transportation distance and time is the biggest barrier to accessing food from a non-profit food hub in the City of Vancouver that survey respondents identified with.

In terms of a corroborating comment from an interview participant, Carmen stressed how non-profit food hubs felt distant and how it took a substantial amount of time to travel to them.

*I think everything was like a bit of ways.. .. Mount Pleasant was like a half an hour transportation ride and Ray-Cam is like maybe forty-five minutes from where we live. The food bank. .. takes a bit longer too. I think half an hour – forty-five minutes – to get to. (Carmen)*

It is important to point out that the amount of time it takes to travel to a non-profit food hub in the City of Vancouver is significant because there are fewer non-profit food hubs in the City of Vancouver than there are other food assets like supermarkets. Unlike middle- and high-income households, low-income households are often not able to afford shopping at other food assets like supermarkets. Although the number of non-profit food hubs in the City of Vancouver is growing, there have been setbacks. For example, near the beginning of the COVID-19 crisis, the Greater Vancouver Food Bank shut down neighbourhood distribution sites that were accessed by hundreds of people on a weekly basis [41]. While increasing the number of non-profit food hubs in the City of Vancouver would provide relief, the deeper issue is the two-tier food system in the City of Vancouver where the economic well-being of a household determines which food assets they can access.

The mode of transportation that interview participants utilized to access a non-profit food hub in the City of Vancouver had a substantial impact on their transportation time. Beyond comments on the difference in transportation time when using an automobile and when using transit, one interview participant with a disability, Kristina, noted how utilizing HandyDART, the local paratransit system for people with disabilities, as their mode of transportation substantially increased the amount of time to access a non-profit food hub in the City of Vancouver, especially because the local paratransit system requires people to be ready for pick-up anytime within a half an hour window.

*Transportation is a big deal.. .. When you travel with HandyDART, it’s a day event. For example,. .. your appointment let’s say is 10 o’clock.. .. You have to be ready at 9 o’clock and. .. they’ve got half an hour to pick you up. So, they pick you up at the end of your window – 9:30 whatever – and you get to your appointment at 10 o’clock. You stay for an hour for your appointment. At 11 o’clock, from 11 to 11:30 is another window. So, it’s kind of a guessing game. (Kristina)*

**Fig. 7:**
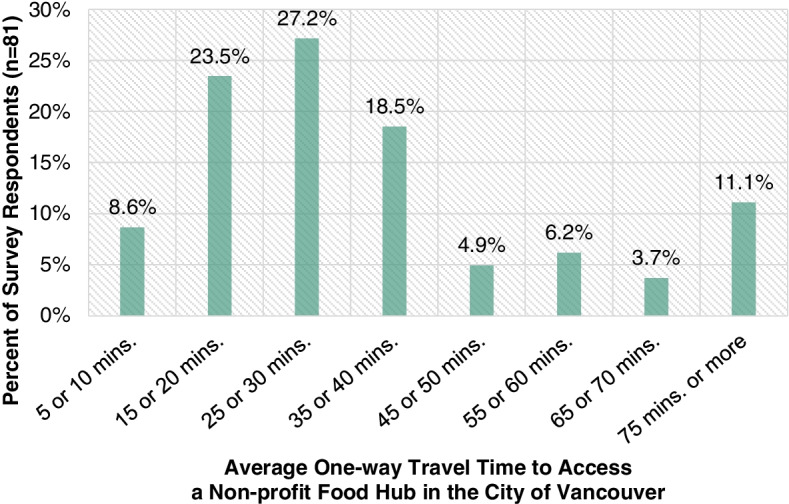
Average one-way travel time for survey respondents to access a non-profit food hub in the City of Vancouver

As highlighted in Fig. 7, the average one-way travel time to access a non-profit food hub in the City of Vancouver varies for survey respondents (*n* = 81). The majority (50.7%) of survey respondents had an average one-way travel time between 10 to 30 mins. However, 44.4% of survey respondents had an average one-way travel time of 35 min or more.

In addition to the grouped barrier of transportation distance and time, there is also the grouped barrier of transportation inconveniences, reliability, and accessibility.

### Transportation inconveniences, reliability, and accessibility

25.3% of survey respondents (*n* = 79) identified with the barrier of transportation inconveniences, 22.8% identified with the barrier of transportation reliability, and 15.2% identified with the barrier of transportation accessibility. Survey respondents were prompted to identify with the barrier of transportation accessibility if they were having issues such as those related to a disability or having to take a stroller for a child. Furthermore, survey respondents were prompted to identify with the barrier of transportation inconveniences if they were having issues such as those related to taking groceries back. The three barriers have been grouped because they are connected. When considering grouped barriers, the barrier of transportation inconveniences, reliability, and accessibility is the second biggest barrier to accessing food from a non-profit food hub in the City of Vancouver that survey respondents identified with.

In terms of a corroborating comment from an interview participant, Sonia stressed how taking groceries back from non-profit hubs is difficult, especially on transit, because the bags are heavy.

*It is an inconvenience to bring the bulk groceries back that I get once a month. It’s just as cumbersome as having a stroller to have to carry two heavy bags of groceries on the bus. (Sonia)*

Another transportation inconvenience brought up in the interviews was particularly relevant to the COVID-19 crisis. An interview participant, Carmen, who has three children, noted how certain fellow transit users would get angry at them for sitting together. It is unclear why certain fellow transit users felt this way, especially since members of the same household have not been required to distance themselves from each other at any point during the COVID-19 crisis. Carmen also noted how certain fellow transit users took issue with her bringing a food cart to help take groceries back. It is disheartening that people like Carmen have to deal with comments regarding a food cart. Furthermore, Carmen noted that a temporary move to rear-door boarding on buses early in the COVID-19 crisis, which was an effort to promote the safety of bus drivers, made it difficult to access food from non-profit food hubs because she had to lift her food cart onto the bus since buses could not lower the rear door [42, 43]. Ultimately, transportation inconveniences and accessibility issues have forced Carmen to rely on non-profit food hubs that deliver food.

*We use a food cart so that takes up space with me and my kids. Like pre-COVID it wasn’t too bad but COVID brought along a lot of barriers.. .. When we were getting onto transit, people would get mad at us for sitting together and then having that cart. When we first started going on transit when COVID hit, it would only be getting on the back, which made it a little difficult pulling the cart on because they can’t lower it. COVID really, really put up a lot of barriers everywhere and we just ended up trying to resource organizations that could deliver food to us. (Carmen)*

Furthermore, another interview participant, Kristina, stressed how the local paratransit system, HandyDART, can be unreliable and cause extremely long waits.

*HandyDART is kind of a nightmare. They could be running late. With buses, if you miss one bus, you know that there’s another bus coming. There’s more flexibility. With HandyDART, you can phone them up and say, “Look my bus hasn’t come, I’m going to skip it and take a bus.” And I’ve done that. I’ve thrown in the towel. I spent three hours once outside of work. .. saying, “Look, I just gotta go, it’s freezing rain and I can’t get back into the office.” And I was on my walker and I couldn’t get back into the building. And they forgot me. And so I had to take a bus and it was a nightmare. I got home around 8 o’clock and I finished at 4:30. (Kristina)*

In addition to the grouped barrier of transportation inconveniences, reliability, and accessibility, there is also the barrier of transportation costs.

### Transportation costs

25.3% of survey respondents (*n* = 79) identified with the barrier of transportation costs. When considering grouped barriers, the barrier of transportation costs is the third biggest barrier to accessing food from a non-profit food hub in the City of Vancouver that survey respondents identified with.

In terms of a corroborating comment from an interview participant, Carmen stressed how transportation costs for herself and three children are high and that it has not made sense for her to pay for monthly transit passes during the COVID-19 crisis because she and her family do not go out often. Yet, Carmen noted that if restrictions related to the COVID-19 crisis were lifted, her family would pay for monthly transit passes and regain the ability to access non-profit food hubs in the City of Vancouver.

*Transit for me and three kids is over $200 a month.. .. Right now, we’re kind of saving on transit because we’re not going out as much. But if the restrictions were lifted, we would go back to monthly and we’d be able to go out and pick up food. (Carmen)*

Another interview participant, Eddie, highlighted how although he is eligible for a free monthly transit pass due to provincial program for people with disabilities, there was a time where he lost access to a free monthly transit pass, which he says is valued at $150, simply because he made $10 more a month as a result of a cost-of-living increase in a federal program. When Eddie lost access to his monthly transit pass, Eddie was not sure about how he would get around and had to rely on a kind couple to help him obtain a monthly transit pass. For those who are not eligible for a free monthly transit pass, especially those who do not have anyone to help them financially, the lack of a monthly transit can be the determining factor behind someone’s inability to access food from a non-profit food hub.

*I’m on the disability so I get a free transit pass so it’s actually very good. There was a time when I got on an increase. I was on CPP disability federal and I would get a top-up from the provincial government but at one time there back in 2011 or 2012, I got a cost-of-living increase that put me 10 dollars over the provincial maximum. I knew I wouldn’t get a top-up but they took my transit pass away, which was really frustrating because how do I get around? Just because I’ve gotten 10 dollars more a month, they’ve taken away my transit pass – which is a 150 dollar a month benefit. So that was tough but I was able to get some help from a really nice couple actually that helped me until such time as the [provincial] NDP gave an increase to people with disabilities. (Eddie)*

In addition to the barrier of transportation costs, there is also the barrier of line-up times at non-profit food hubs.

### Line-up times at non-profit food hubs

17.7% of survey respondents (*n* = 79) identified with the barrier of line-up times at non-profit food hubs. When considering grouped barriers, the barrier of line-ups at non-profit food hubs is the fourth biggest barrier to accessing food from a non-profit food hub in the City of Vancouver that survey respondents identified with.

In terms of a corroborating comment from an interview participant, Belen noted that as a result of long line-up times at non-profit food hubs, especially during the COVID-19 crisis, she had to ensure that she got there early to avoid having to wait in line.

*Especially during COVID, it’s the line-ups. Which is why I try to get there by 9 o’clock, which is as soon as they open.. .. I have no choice but to go because nobody wants to run out of groceries and that’s the only place where we can get affordable [food] on our low-income. (Belen)*

**Fig. 8:**
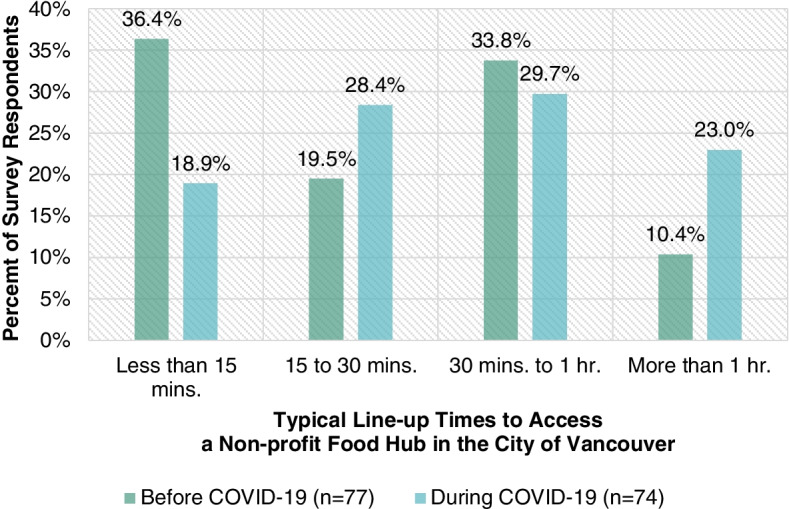
Typical line-up times survey respondents face to access a non-profit food hub in the City of Vancouver

As highlighted in Fig. 8, the typical line-up time to access a non-profit food hub in the City of Vancouver varies for survey respondents both before (*n* = 77) and during (*n* = 74) the COVID-19 crisis. The percent of survey respondents who had a typical line-up time that was less than 15 min dropped from 36.4% before the COVID-19 crisis to 18.9% during the COVID-19 crisis. Furthermore, the percent of survey respondents who had a typical line-up time that was greater than an hour rose from 10.4% before the COVID-19 crisis to 23% during the COVID-19 crisis.

In addition to the barrier of line-up times at non-profit food hubs, there is also the barrier of schedules of non-profit food hubs.

### Schedules of non-profit food hubs

15.2% of survey respondents (*n* = 79) identified with the barrier of line-up times at non-profit food hubs. When considering grouped barriers, the barrier of schedules at non-profit food hubs is the fifth biggest barrier to accessing food from a non-profit food hub in the City of Vancouver that survey respondents identified with.

In terms of a corroborating comment from an interview participant, Irene stressed how the schedules of non-profit food hubs in the City of Vancouver are a barrier to accessing food from them since she has to work and is only able to access them after work. Irene recounted times where she was coming home from work and was stressed out because she was not sure if she would make it to the non-profit food hub in time.

*I have to make sure that I get there. I typically go sort of later in the day. Usually, it would be after my work time. So there have been times where when I am coming home from work, I am stuck on the bus and there is some stress involved – whether I am going to be back in time to pick up my food that day. (Irene)*

Irene’s comments align with how some non-profit food hubs in the City of Vancouver are scheduled. For example, the Greater Vancouver Food Bank’s branch in the City of Vancouver is only open from 10 AM to 4 PM on Tuesdays, Thursdays, and Fridays [44]. Those who work typical office hours may only be able to access food from the Greater Vancouver Food Bank when they are open from 1 PM to 7 PM on Wednesdays and/or from 10 AM to 2 PM on Saturdays [44]. Furthermore, some non-profit food hubs like the Grandview Woodland Food Connection only have food available for pickup once a month on a particular day [45]. This is quite different from your average supermarket which is typically open every day from 8 AM to 9 PM. It is important to consider that in one study which surveyed 340 food banks, approximately three-quarters only allowed people to obtain food assistance once per month, which is not sufficient [46].

Irene is not the only interview participant who finds the schedules of non-profit hubs in the City of Vancouver to be a barrier. For example, Paula mentioned that there are months where her work schedule conflicts with the day and time that the non-profit food hub she accesses has food available for pickup that month. On those months, she cannot access food from the non-profit food hub.

*There’s an occasional time where if the time conflicts with my work schedule. .. then I can’t attend that month. But then if I’m able to, I will take the food. (Paula)*

## Discussion

The survey respondents in this study identified with several barriers to physical and economic access to food when attempting to access food from non-profit food hubs in the City of Vancouver. Furthermore, comments from interview participants corroborate the various barriers. In this section, recommendations are provided regarding what can be done to promote better and dignified access to food. The recommendations are categorized into interventions focused on improving physical access to food and interventions focused on improving economic access to food.

### Interventions focused on improving physical access to food

The first recommendation focused on improving physical access to food is that non-profit food hubs should maintain a food delivery option. When considering grouped barriers, the barrier of transportation distance/time and the barrier of transportation inconveniences/reliability/accessibility were the two biggest barriers that survey respondents identified with in terms of accessing food from a non-profit food hub in the City of Vancouver. Maintaining a food delivery option would mean that users of non-profit food hubs in the City of Vancouver, especially vulnerable populations, will not have to physically go to a non-profit food hub to access food. When individuals and families have to physically go to a non-profit food hub in the City of Vancouver, the one-way travel time can sometimes be 35 min or more. Furthermore, many individuals and families struggle to physically get there because it is difficult to carry groceries and/or to work around accessibility issues. Although the COVID-19 crisis has been a troubling time for many of those who access food from non-profit food hubs in the City of Vancouver, many appreciate those non-profit food hubs which have implemented a food delivery option. Maintaining a food delivery option would also address other barriers to accessing food from a non-profit food hub in the City of Vancouver such as transportation costs, line-up times at non-profit food hubs, and schedules of non-profit food hubs. With fewer physical and economic barriers, low-income individual individuals and families would enjoy greater food access and food security.

The second recommendation focused on improving physical access to food is that the local transportation authority (TransLink) should provide convenient and reliable paratransit service. HandyDART is the local paratransit system for people with disabilities. The door-to-door service provided by HandyDART is extremely valuable for those who find it difficult to navigate conventional public transit. When considering grouped barriers, the barrier of transportation inconveniences/reliability/accessibility was the second biggest barrier that survey respondents identified with in terms of accessing food from a non-profit food hub in the City of Vancouver. Some survey respondents indicated that they use HandyDART to access food from non-profit food hubs in the City of Vancouver and some interview participants noted how HandyDART was cumbersome and/or unreliable. Those who struggle to carry groceries and/or work around accessibility issues would benefit from convenient and reliable access to HandyDART service. TransLink can make HandyDART more convenient and reliable by shortening pick-up time windows and by having more buses and drivers such that the service is fast and such that there is no need for drivers to drive away prematurely. The HandyDART Modernization Program has been seeking to improve the customer experience from start to finish, including how people register, how they book their trips, and how they pay for the service [47]. TransLink mentions that the slated improvements will allow for increased flexibility and the ability to make more spontaneous trips [47]. However, TransLink needs to ensure that the new registration process does not become an effort to deny service to certain people and/or make it more difficult to register for HandyDART service.

The third recommendation focused on improving physical access to food is that the provincial government should consider subsidizing transit passes for low-income households. When considering grouped barriers, the barrier of transportation costs was the third biggest barrier that survey respondents identified with in terms of accessing food from a non-profit food hub in the City of Vancouver. Subsidizing transit passes would help promote the financial security of low-income households. At present, TransLink allows a maximum of four children under the age of five to accompany a paying guardian on transit for free and no one else can use transit for free [47]. However, the provincial government allocated funding to public transit authorities to ensure that all children under the age of twelve can use transit for free starting in September 2021 [48]. This welcome change will allow families with youth to save up to $672 per child annually [49]. In addition, through the BC Bus Pass Program, the provincial government provides a completely subsidized transit pass to people with disabilities and gives access to a discounted transit pass to low-income seniors and other eligible people [50]. The completely subsidized transit pass for people with disabilities is an annual pass that can be denied in favour of receiving $52 each month as a transportation supplement [51]. The discounted transit pass for low-income seniors and other eligible people is an annual pass that costs $45 [52]. In the absence of any transit pass program, a monthly adult transit pass would cost between $98 and $177 a month, or $1176 and $2121 a year, depending on the number of transit zones it is for [49]. Meanwhile, a monthly concession transit pass, available to HandyCard holders, seniors 65 years old and older, and children 5 to 18 years old would cost $56 a month, or $672 a year, regardless of the number of transit zones [49]. Considering how much TransLink charges, ineligibility for the BC Bus Pass Program can significantly impact the financial security of low-income households, especially for those with multiple household members. Therefore, the BC Bus Pass Program could be expanded to cover low-income households. It is worth noting that the BC Bus Pass Program does not include access to HandyDART and including access to HandyDART would help address the barrier of transportation inconveniences/reliability/accessibility [50]. It is important to note that simply providing access to transit is not enough because the barriers associated with food access go beyond the cost of transit.

### Interventions focused on improving economic access to food

In addition to interventions focused on improving physical access to food, there are interventions focused on improving economic access to food that can tackle the root causes of poverty, which is income related.

This first recommendation focused on improving economic access to food is that the provincial and/or federal governments should consider bolstering existing government assistance programs. Bolstering existing government assistance programs would help promote the financial security of low-income households. In this study, 29.6% of survey respondents (*n* = 81) had a total household income of less than $30 k in the past 12 months including any income from government assistance programs. Having a low income and a high cost of living can create immense financial stress for individuals and families. The Canadian Rental Housing Index notes that renters in the lowest income group in the City of Vancouver, those who make between $0 and $23,605 a year with an average of $12,939 a year, typically spend 91% of their household income on rent and utilities [53]. Furthermore, the Canadian Rental Housing Index notes renters in the second-lowest income group in Vancouver, those who make $23,605 to $50,389 with an average of $36,782 a year, typically spend 38% of their household income on rent and utilities [53]. When a household spends 50% or more of their household income on rent and utilities, housing is considered severely unaffordable, and this is the case with those in the lowest income group in the City of Vancouver [53]. Food banks across the country, year over year, note that many come to food banks because they would not otherwise have economic access to food due to the high cost of housing [39]. The COVID-19 crisis has exacerbated the financial stress of low-income households in the City of Vancouver. Whereas 43.9% of survey respondents’ households found it very difficult or difficult to make ends meet including any government assistance programs before the COVID-19 crisis, 67.5% did during the COVID-19 crisis. Promoting the financial security of low-income individuals and families will address the root issue of poverty and will not be a stop-gap solution. At present, there is essentially a two-tier food system in the City of Vancouver where middle- and high-income households have access to much more food than low-income households. If the amount of money that low-income individuals and families receive through welfare and other social assistance programs is increased, they will have the ability to spend that money on what is truly adequate food for them since they will be able to afford shopping at the same food assets that middle- and high-income households’ access. Such an approach is related to food sovereignty and does more to challenge a top-down, charity-model approach to tackling food insecurity, where community members are not provided with extensive freedom of choice (e.g., food banking). It would also help prevent people from having to face the stigma associated with food banking.

The second recommendation focused on improving physical access to food is that the federal government should consider implementing a universal basic income. A universal basic income is “an income paid by a political community to all its members on an individual basis, without means test or work requirement” ([54] p8). Implementing a universal basic income would help promote the financial security of low-income households. However, it is important to note that a universal basic income that is large enough to live on and that does not have phaseout or other eligibility restrictions has never been implemented in a rich country on a large scale or even in a pilot experiment [55]. Furthermore, it is important to be sensitive to concerns that implementing a universal basic income would lead to unsustainable debt and/or inflation. Direct payments by governments during the COVID-19 crisis for some eligible residents have raised renewed interest in a universal basic income [56]. The implementation of a universal basic income could allow individuals and families to decide what to spend money on, rather than having the government provide a wide array of social assistance programs [57]. As with the recommendation of bolstering existing government assistance programs, this approach is related to food sovereignty and challenges a top-down or charity-model approach to tackling food insecurity, where community members are not provided with extensive freedom of choice (e.g., food banking). It would also help prevent people from having to face the stigma associated with food banking. A universal basic income may be better than government assistance programs in that it removes disincentives to unlimited economic participation that are created through eligibility criteria. Furthermore, a universal basic income may be better than government assistant programs in that it empowers low-income individuals and families to save and invest capital in order to rise above the poverty level.

#### Limitations

One limitation of the study is the small sample size. Only 84 completed survey responses were received. Given the small sample size, the findings from the online survey may not be representative of the population that fits the inclusion/exclusion criteria. For example, a large proportion of survey respondents identified as White and this does not align with the reality that Indigenous people and visible minorities, particularly Black people, disproportionately experience food insecurity in Canada [4]. Unfortunately, due to the difficulty of estimating the size of the population that fits the inclusion/exclusion criteria, margins of error for the findings from the online survey were not calculated. The difficulty of estimating the size of the population that fits the inclusion/exclusion criteria is the result of the broad range of food assets that come under the umbrella of non-profit food hubs and a lack of data on these food assets.

Another limitation of the study is uncertain data reliability. Although this study was initially going to proceed with the use of in-person surveys at non-profit food hubs in the City of Vancouver, which would have provided a greater level of certainty in terms of data reliability, public health concerns around conducting in-person surveys during the COVID-19 crisis meant the study had to proceed with the use of an online survey. As with many online surveys, especially ones where survey respondents are offered a monetary reward in exchange for their involvement, certain survey respondents may have claimed to fit the inclusion/exclusion criteria even though they did not. It is also possible that certain survey respondents made multiple completed survey responses. Furthermore, certain survey respondents may have provided inaccurate information.

An additional limitation of the study is survey and interview inaccessibility. Since the study utilized an online survey that was only available in English, certain populations groups may not have been able to participate in the survey, such as those who did not have access to a suitable device with an internet connection, those who were not proficient in the use of technology, those who were unable to read English well, and those with certain disabilities. For example, a large proportion of survey respondents were young adults, and this may have been influenced by how an online survey may not be accessible to those older adults who are not proficient in the use of technology. Furthermore, if an individual did not have an email on file with the BC Poverty Reduction Coalition or one of the non-profit food hubs in the City of Vancouver which helped disseminate the link to the online survey, if they did not come across the link via social media or the BC Poverty Reduction Coalition’s website, and if they did not otherwise come across the link via an individual or organization, the online survey would have been inaccessible. In addition to the accessibility limitations of the online survey, since the online survey requested survey respondents interested in participating in a key informant interview to leave their email address or phone number, certain populations groups may not have been able to participate in the interviews, such as those who did not have an email address or a phone with service. Furthermore, since potential interview participants were given the choice of having the interview done over the phone or an online meeting, certain populations groups may not have been able to participate in the interviews, such as those who did not have access to a phone with service or a suitable device with an internet connection.

## Conclusion

Despite attempts to end hunger and achieve food security, there is a growing number of people affected by food insecurity [2–4]. The COVID-19 crisis has only exacerbated the issue of food insecurity globally and in Canada [2, 5]. Although the Government of Canada attempts to secure the right to adequate food through social assistance and unemployment insurance programs, these programs have been inadequate. The inadequacy of these programs has made it necessary for food banks and other non-profit organizations to fill the gap [8]. While food banks and other non-profit organizations are certainly beneficial, they are not the best solution in terms of securing the right to adequate food. Although clients of food banks and other non-profit organizations may be treated well, they are forced to accept food that is not always sufficient, nutritionally balanced, or otherwise adequate [10]. Currently, solutions to address urban food insecurity in Canada are focused on improving existing food assets in cities and/or growing the number of food assets in cities. While this addresses the food security dimension of food availability, it does not address the food security dimension of food access. This study evaluated access to food at non-profit food hubs in the City of Vancouver before and during the COVID-19 crisis, especially as it relates to mobility and access to transportation. The findings from this study include identification of which individuals and families access non-profit food hubs in the City of Vancouver, how they access the non-profit food hubs, and what barriers they face to access the non-profit food hubs. To address the prevalent barriers that users of non-profit food hubs in the City of Vancouver face, this study recommends that non-profit food hubs maintain a food delivery option and that the local transportation authority (TransLink) provides convenient and reliable paratransit service. Furthermore, this study recommends that the provincial government considers subsidizing transit passes for low-income households, that the provincial and/or federal governments consider bolstering existing government assistance programs, and that the federal government considers implementing a universal basic income. We need to move from a top-down, charity-model approach to tackling food insecurity to a food sovereignty approach to tackling food insecurity. It is time to deconstruct the two-tier food system.

## Data Availability

The datasets used and/or analyzed during the current study are available from the corresponding author on reasonable request.
